# Evidex

**DOI:** 10.5195/jmla.2019.587

**Published:** 2019-04-01

**Authors:** Meghan A. Hupe

**Affiliations:** Director of Information and Delivery Services, Dahlgren Memorial Library, Georgetown University, Washington, DC, maw55@georgetown.edu

## OVERVIEW

The health care informatics company Advera Health Analytics launched Evidex in 2016. The database provides information on drug safety and adverse events. According to Advera Health Analytics, “With evidence of a drug’s safety and efficacy constantly evolving, our clients need to gather data quickly, conduct comprehensive analysis and make standardized comparisons to inform recommendations and decisions. Evidex was designed from the ground up to fill this important data and analytics gap” [1].

Users can search this resource for individual drugs or compare their differences in costs, outcomes, safety, efficacy, and more. The Food and Drug Administration (FDA) Adverse Event Reporting System (FAERS) is integrated into Evidex. Advera Health Analytics reports, “Evidex is the first web based platform that combines annotated data from curated clinical trial results with structured real world drug evidence” [1]. The clinical trials results are selected from ClinicalTrials.gov and other published research with “over 2,000 drugs covered in the database and over 800 clinical trials” [1].

Advera Health Analytics states that “2018 WILL BE the year that the pharmacovigilance industry embraces the next generation of drug safety data, analytics, and software, what we call Pharmacovigliance 2.0” [2]. Pharmacovigilance is the collection, detection, assessment, monitoring, and prevention of adverse events with pharmaceutical products, which typically applies to safety departments in pharmaceutical companies. According to Advera Health Analytics, its:

proprietary clinical trials safety outcomes database, when linked with optimized post-approval spontaneous reporting data from the FDA Adverse Event Reporting System (FAERS) and Vigibase, claims data and social media provides pharmacovigilance professionals with software, data and analytics to track emerging safety issues through multiple data sets, validate signals seen in spontaneous reporting and engage across these various data sets in dynamic and proactive manner. [3]

Evidex is a subscription-based service. Institutional users are required to create a personal account and, once created, will receive email confirmation from the institution’s assigned Evidex product specialist, along with login procedures and the product specialist’s contact information. Occasionally, the Evidex product specialists will send targeted emails to subscribers, such as updates on the database, and can create customized promotional materials.

## MAJOR FEATURES

Once logged into the database, the user sees a familiar Google-like layout and can conduct a broad search using keywords. Also available is a drop-down menu with the following categories: drugs, companies, indications, classes/mechanisms of action, adverse events, and adverse events analysis. Auto-suggest displays terms by category, then narrows down results as the user types, and is useful when the user is uncertain about the correct spelling of a drug name. It also provides the generic as well as the brand name of a drug. For librarians or experienced searchers, a feature called “Custom Drug Analysis” provides a custom search option that is conveniently located below the search box.

On the results page, the user will find comprehensive information about a particular drug. At the top of the results page, the drug name is listed along with its generic and brand name. To the right is a “Boxed Warning” (if applicable) and “RxScore,” which is a 1–100 scale that represents an overall assessment of a drug’s risk to a patient: the lower the score, the safer the drug is with less serious side effects.

Underneath the drug name are several tabs: Overview, Alerts, FAERS, Clinical, VigiBase, Social, and Claims. The Overview section includes useful information such as the date when the FDA approved the drug, how many drug names are associated with the specific drug, and when the information on the drug was last updated. It also includes the drug’s classification and indications. The Alerts tab provides information on any important updates about the drug, such as label changes. The FAERS tab includes a report on a drug’s adverse events, cost burden, safety score, outcomes, cases, and a summary of the information. It is important to note that a detailed chart is provided under Adverse Events, where adverse events are listed along with an RxSignal, cases, reports, serious events, and literature references. RxSignal is proprietary tool of Advera Health Analytics that monitors the adverse events that are not listed on a drug’s label, potentially triggering a label change if there is an increased rate of reports.

The Clinical feature provides clinical results through ClinicalTrials.gov, although some drugs might not have clinical trials available. Vigibase, Social, and Claims features require an additional subscription cost and are geared toward pharmaceutical and insurance companies.

Evidex has integrated a World Health Organization database, Vigibase, which generates global case safety reports and is administered by Uppsala Monitoring Centre in Sweden. Vigibase is a:

pharmacovigilance database, in which information is recorded in a structured, hierarchical form that allows flexible and easy retrieval and analysis of the data. Its purpose is to provide the evidence from which potential medicine safety hazards (signals) may be detected and communicated. [4]

Advera Health Analytics notes that:

While FAERS pulls in adverse events reports from patients outside the U.S., it is highly concentrated by U.S. medications and submissions by U.S. patients and healthcare providers. Adding the Vigibase data allows a more global perspective on medication use and patient safety with approximately 15 million reports from over 100 countries that cover 90% of the world’s population. [5]

The Social feature monitors social media for drug side effects, and the Claims feature tracks emerging safety issues through electronic health records (EHRs), claims, and clinical trials. Advera Health Analytics can work with the subscriber’s EHR provider to potentially integrate data into their analytics.

The Adverse Events search results page presents information on cases reported to have an adverse event as well as reports on top drugs and their indications for adverse events. Users will find detailed charts and descriptions of different terms that are similar to the results for drugs.

Upon request, Advera Health Analytics can provide institutions with user statistics, although these are not COUNTER compliant. The statistics provide information on page use and the number of users accessing the database. This information can be useful when institutions make decisions on renewing their subscriptions with the database.

## AUDIENCE

According to Advera Health Analytics, this tool is intended for a variety of users including pharmaceutical companies, health insurers, hospitals, academic institutions, and financial institutions. Pharmaceutical companies might utilize Evidex to understand comparative safety issues in FDA-approved drugs, while health insurers and hospitals could use this database for clinical evidence, costs, and safety data to ensure better decisions for patients. Academic institutions and libraries can access this database to conduct research on clinical and safety issues for potential further study.

## USABILITY

Generally, Evidex requires additional training for those who are not familiar with the different types of statistical information on the result pages. If questions arise, users can call, email, chat, or go to Evidex’s Knowledge Base, where product searching assistance is available. A chat icon is conveniently located on each web page, and the support team responds in a timely manner. The Knowledge Base is a brief guide on how to use the different features of the product and provides definitions, methodologies, and images of the product. Users can also request a webinar with their assigned product specialist for a more in-depth overview of the resource. In addition, users can “hover” over each column header on results pages to access pop-up descriptions.

A search box is displayed on every results page to make it simple for users to search for a drug or adverse event immediately. While it is convenient to have the search box on each page, this reviewer noted problems where results would not display for the new search, and the reviewer had to return to the home page.

All literature references in Evidex are linked to PubMed, and Advera can work with institutions to connect the PubMed link to specific holdings. This reviewer found that while searching in this database, the results pages were slow to load and explanations for some of the data were not provided. For example, on the results page of drug adverse effects, a definition button is displayed, but there was no information provided. There was also no explanation for the RxScore, leaving users who are unfamiliar with Evidex wondering what it represents.

## CONCLUSION

Evidex is an efficient Google-like platform that offers an abundance of information about and analysis of drug safety and adverse events using FAERs and ClinicalTrials.gov to curate results. Custom Drug A-E Analysis is an excellent advanced feature for librarians to use to focus their searches, while the auto-suggest assists in a quick, simple search. Overall, this database is easy to navigate, and the explanations of the charts and terms are excellent and comprehensive.
